# Supplementary material for: SARS-CoV-2 nsp1 mediates broad inhibition of translation in mammals
Source: bioRxiv. 2025 Jan 15:2025.01.14.633005. Preprint. [Version 1] doi: 10.1101/2025.01.14.633005 (PMC11761087; doi:10.1101/2025.01.14.633005)
Supplement: 1 [file NIHPP2025.01.14.633005v1-supplement-1.pdf]

bioRxiv preprint doi: https://doi.org/10.1101/2025.01.14.633005; this version posted January 15, 2025. The copyright holder for this preprint (which was not certified by peer review) is the author/funder, who has granted bioRxiv a license to display the preprint in perpetuity. It is made available under aCC-BY 4.0 International license.

Supplementary Materials

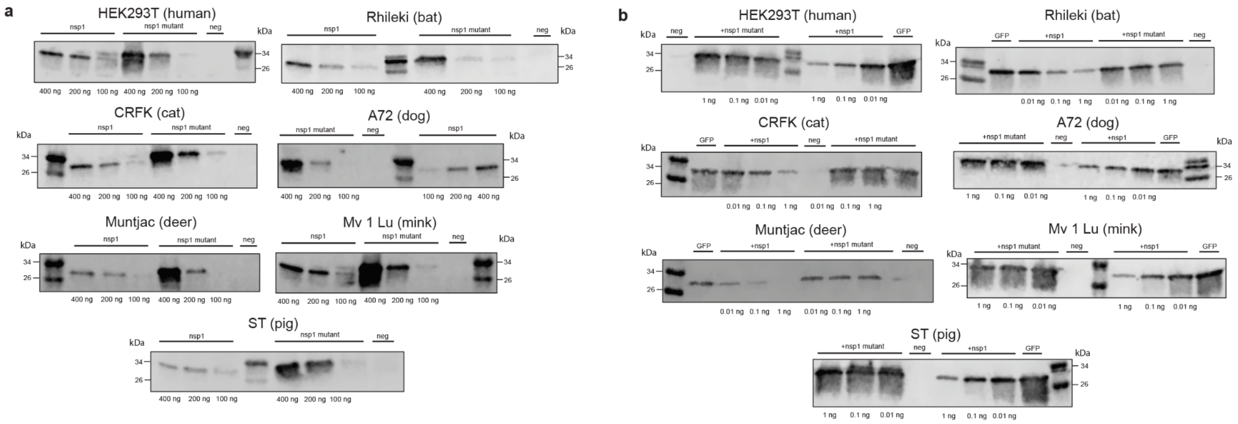

Supplementary Figure S1. Protein expression in animal cell lines, related to Figures 1 and 3. (a) Western blots of nsp1 and nsp1 mutant expression using anti-nsp1 polyclonal antibody (Thermo) in seven different animal cell lines with transfection amounts listed below the images. Transfection assays for these western blots were performed in 12-well plates with increased amounts of mRNA to enable detection of expression of the wildtype and of the K164A/H165A mutant nsp1 constructs, which could not be detected in a 96-well format (with much lower amounts of mRNA transfected) used for Figures 1 and 3. (b) Western blot analysis of GFP expression in the presence or absence of the wildtype or the K164A/H165A mutant nsp1 shown in Figures 1 and 3 using anti-GFP monoclonal antibody GF28R (Thermo). Neg: negative control.

Supplementary Figure S2. Purification of the bat Rhileki ribosome and SARS-CoV-2 nsp1, related to Figure 2.

bioRxiv preprint doi: https://doi.org/10.1101/2025.01.14.633005; this version posted January 15, 2025. The copyright holder for this preprint (which was not certified by peer review) is the author/funder, who has granted bioRxiv a license to display the preprint in perpetuity. It is made available under aCC-BY 4.0 International license.

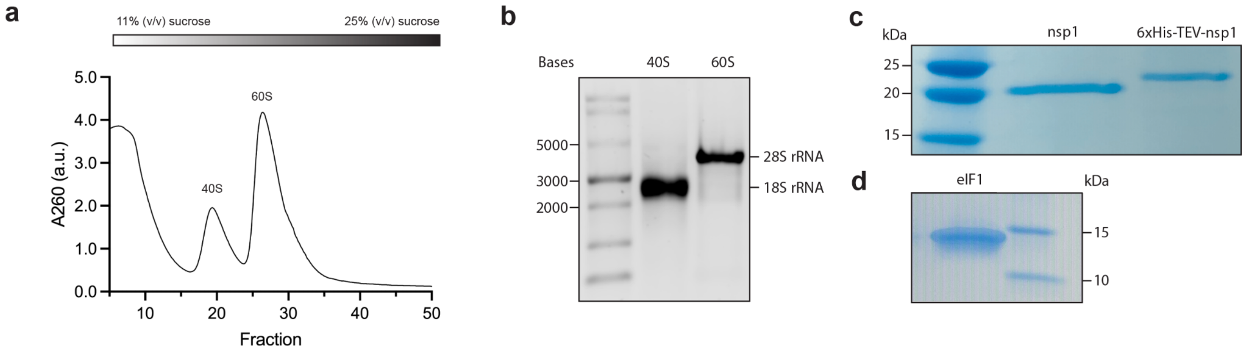

(a) Isolation of the Rhileki ribosomal 40S and 60S subunits using a 11-25% (v/v) sucrose density gradient. The purification process was followed using absorbance at 260 nm (A_{260}) of each fraction. (b) 1% bleach agarose gel showing separation of the 40S and 60S ribosomal subunits through 18S and 28S rRNA respectively. An RNA ladder was used to deduce separation of the 18S and 28S bands. (c) SDS-PAGE gel of recombinant SARS-CoV-2 nsp1 with or without the 6xHis-TEV tag. (d) SDS-PAGE gel of recombinant human eukaryotic initiation factor 1 (eIF1). TEV: Tobacco Etch Virus protease cleavage site.

bioRxiv preprint doi: https://doi.org/10.1101/2025.01.14.633005; this version posted January 15, 2025. The copyright holder for this preprint (which was not certified by peer review) is the author/funder, who has granted bioRxiv a license to display the preprint in perpetuity. It is made available under aCC-BY 4.0 International license.

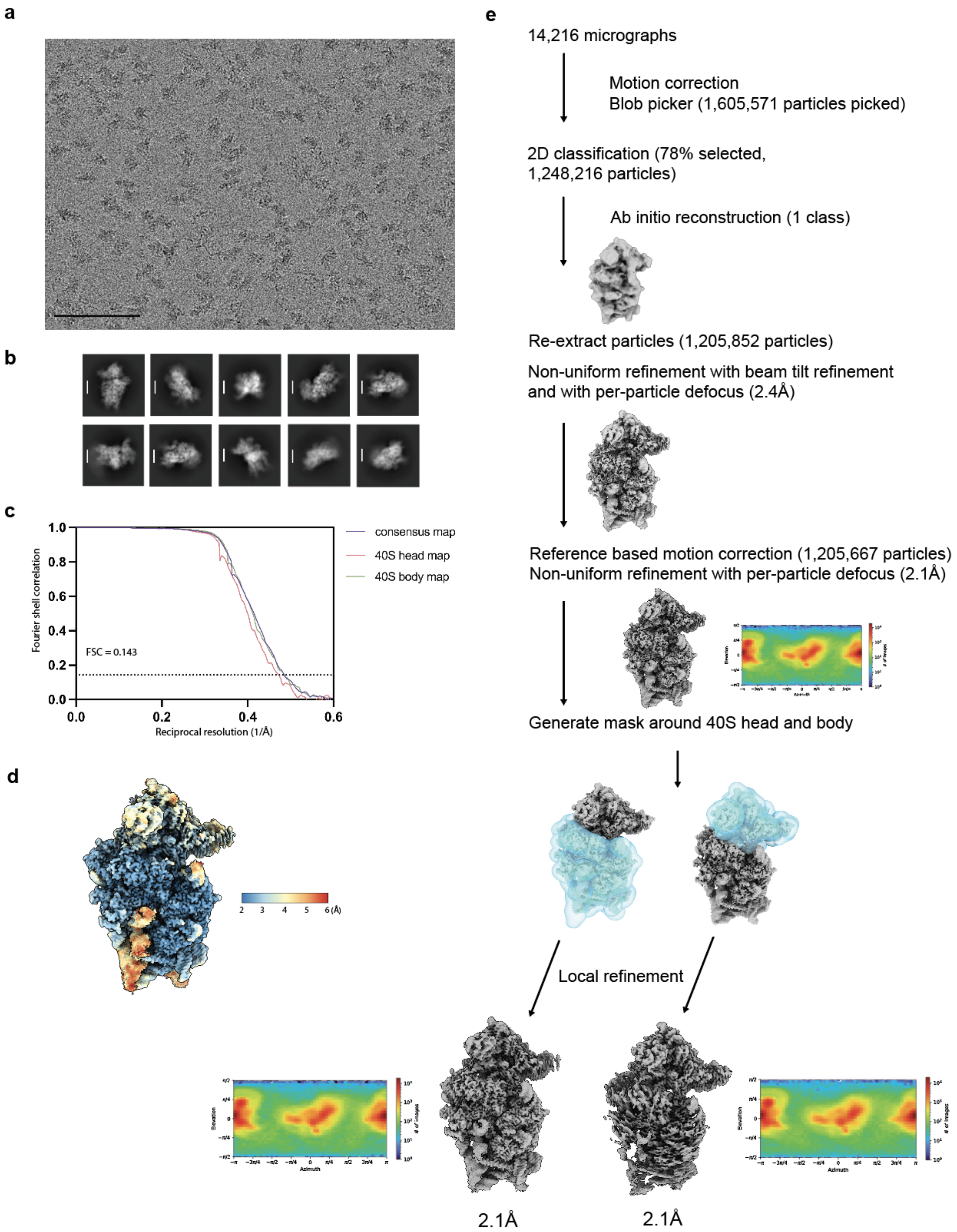

Supplementary Figure S3. Cryo-EM workflow of SARS-CoV-2 nsp1 bound 40S Rhileki ribosome structure, related to Figure 2. (a) Representative electron micrograph and (b) 2D class averages. Scale bars of the micrograph and class averages are 100 nm and 100Å, respectively. (c)

bioRxiv preprint doi: https://doi.org/10.1101/2025.01.14.633005; this version posted January 15, 2025. The copyright holder for this preprint (which was not certified by peer review) is the author/funder, who has granted bioRxiv a license to display the preprint in perpetuity. It is made available under aCC-BY 4.0 International license.

Combined gold-standard fourier shell correlation (FSC) curves for the consensus map (purple line) and the locally refined maps, separated for the 40S head (pink line) and body (green line). (d) Local resolution map of the consensus refinement. (e) Data processing flowchart for the reconstruction of the consensus map and the subsequent locally refined maps. Angular distribution plots with all the particles contributing to the final maps are shown for the three maps.

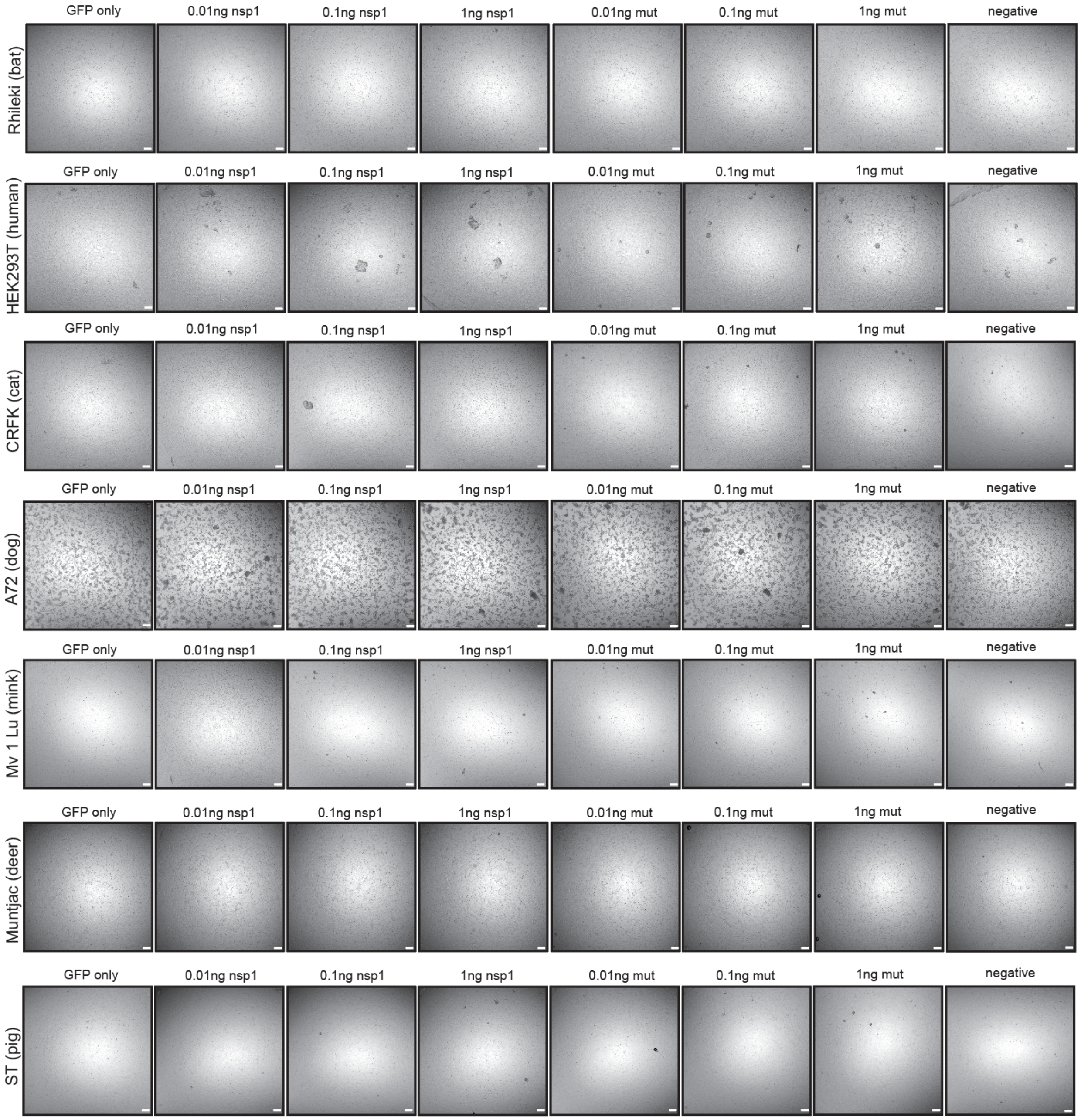

Supplementary Figure S4. Bright-field images of cell lines used for transfections, related to Figures 1 and 3. Corresponding bright-field images of fluorescence images. Negative: non-transfected cells. Scale bar: 100 µm.

bioRxiv preprint doi: https://doi.org/10.1101/2025.01.14.633005; this version posted January 15, 2025. The copyright holder for this preprint (which was not certified by peer review) is the author/funder, who has granted bioRxiv a license to display the preprint in perpetuity. It is made available under aCC-BY 4.0 International license.

Table S1: Cryo-EM data collection, refinement, and validation statistics, related to Figure 2 and Figure S3

	Rhileki ribosome consensus PDB ### EMD ###	Rhileki ribosome 40S body PDB ### EMD ###	Rhileki ribosome 40S head PDB ### EMD ###
**Data collection and processing**			
**Magnification**	105,000		
**Voltage (kV)**	300		
**Electron exposure (e–/Å^2)**	30.7		
**Defocus range (µm)**	-0.6 - -1.6		
**Pixel size (Å)**	0.829		
**Symmetry imposed**	C1		
**Initial particle images (no.)**	1,605,571		
**Final particle images (no.)**	1,205,667		
**Map resolution (Å)**	2.1		
**FSC threshold**	0.143		
**Refinement**			
**Model resolution (Å)**	2.2	2.2	2.4
**FSC threshold**	0.5	0.5	0.5
**Map sharpening *B* factor (Å^2)**	55.1	56.7	59.2
**Model composition**			
**Non-hydrogen atoms**	52405	56113	25366
**Protein residues / Nucleotides**	3237 / 1218	3087 / 1281	1897 / 483
**Ligands**	72	83	32
**Water**	1639	1640	826
***B* factors (Å^2)**			
**Protein**	82.66	89.4	46.3
**Nucleotide**	92.92	108.6	32.7
**Ligand**	76.84	83.6	29.0
**Water**	67.21	78.9	26.0
**R.m.s. deviations**			
**Bond lengths (Å)**	0.003	0.006	0.003
**Bond angles (°)**	0.508	0.579	0.594

bioRxiv preprint doi: https://doi.org/10.1101/2025.01.14.633005; this version posted January 15, 2025. The copyright holder for this preprint (which was not certified by peer review) is the author/funder, who has granted bioRxiv a license to display the preprint in perpetuity. It is made available under aCC-BY 4.0 International license.

**Validation**			
**MolProbity score**	1.26	1.37	1.55
**Clashscore**	4.95	6.00	5.64
**Poor rotamers (%)**	0.57	1.15	1.60
**Ramachandran plot**			
**Favored (%)**	98.89	99.11	97.57
**Allowed (%)**	1.11	0.89	2.43
**Disallowed (%)**	0.00	0.00	0.00

Table S2: Model contents and sequences used for structure building, related to Figure S3

Name	Alternative Name	Chain ID	Length (residues)	Modeled residues	UniProt ID (proteins)
40S Ribosomal RNA / Protein					
18S rRNA		i	1869	1-232,287-689,745-751,792-1869	NA
S2	uS5	A	293	58-278	NA
S3	uS3	B	243	4-227	NA
S3a	eS1	C	264	2-8,23-232	NA
S4	eS4	D	263	2-261	NA
S5	uS7	E	204	15-129,133-204	NA
S6	eS6	F	249	1-231	NA
S7	eS7	G	194	5-108,111-194	NA
S8	eS8	H	208	2-206	NA
S9	uS4	I	194	2-181	NA
S10	eS10	J	165	1-96	NA
S11	uS17	K	158	3-24,31-153	NA
S12	eS12	K	132	12-92, 102-128	NA
S13	uS15	M	151	2-151	NA
S14	uS11	N	151	18-151	NA

bioRxiv preprint doi: https://doi.org/10.1101/2025.01.14.633005; this version posted January 15, 2025. The copyright holder for this preprint (which was not certified by peer review) is the author/funder, who has granted bioRxiv a license to display the preprint in perpetuity. It is made available under aCC-BY 4.0 International license.

S15	uS19	O	145	13-136	NA
S15a	uS8	P	130	2-130	NA
S16	uS9	Q	146	7-146	NA
S17	eS17	R	135	2-132	NA
S18	uS13	S	152	2-145	NA
S19	eS19	T	145	3-143	NA
S20	uS10	U	119	17-116	NA
S21	eS21	V	83	1-83	NA
S23	uS12	W	143	2-141	NA
S24	eS24	X	132	4-128	NA
S25	eS25	Y	125	42-112	NA
S26	eS26	Z	115	2-99	NA
S27	eS27	a	84	2-84	NA
S27a	eS31	b	156	80-150	NA
S28	eS28	c	69	7-68	NA
S29	uS14	d	56	2-56	NA
S30	eS30	e	133	78-119, 126-132	NA
L41	eL41	f	25	1-22	NA
RACK1		g	317	3-274,280-314	NA
RPSA	LRP	h	295	2-213	NA
SARS-CoV-2 nsp1		j	180	148-180	P0DTC1
eIF1		k	113	30-48,54-76,81-100,110-112	P41567
